# Lack of Acute Toxicity and Mutagenicity from Recombinant *Epinephelus lanceolatus* Piscidin Expressed in *Pichia pastoris*

**DOI:** 10.3390/md18040206

**Published:** 2020-04-11

**Authors:** Hsiao-Ching Chen, Chieh-Yu Pan, Venugopal Rajanbabu, Yen-Yun Lee, Wei-Ren Tsai, Jyh-Yih Chen

**Affiliations:** 1 Division of Applied Toxicology, Taiwan Agricultural Chemicals and Toxic Substances Research Institute, Council of Agriculture, Taichung City 41358, Taiwan; schingc@tactri.gov.tw (H.-C.C.); lyy@tactri.gov.tw (Y.-Y.L.); 2 Department and Graduate Institute of Aquaculture, National Kaohsiung University of Science and Technology, Kaohsiung 811, Taiwan; panjade@nkust.edu.tw; 3 Anbil Dharmalingam Agricultural College and Research Institute, Tamil Nadu Agricultural University, Tiruchchirapalli, Tamil Nadu 620027, India; vrajanbabu@gmail.com; 4 Marine Research Station, Institute of Cellular and Organismic Biology, Academia Sinica, 23-10 Dahuen Road, Jiaushi, Ilan 262, Taiwan; 5 The iEGG and Animal Biotechnology Center, National Chung Hsing University, Taichung 402, Taiwan

**Keywords:** *Epinephelus lanceolatus* piscidin, antimicrobial peptide, recombinant piscidin, toxic effects, allergic effects, mutagenetic toxicity

## Abstract

The antimicrobial peptide (AMP) piscidin was identified from *Epinephelus lanceolatus* and demonstrated to possess antimicrobial and immune-related functions. Supplementation of feed with recombinant *Epinephelus lanceolatus* piscidin (rEP)-expressing yeast pellets may minimize the excessive use of antibiotics and control pathogens in aquaculture or animal husbandry. However, before implementing rEP as a supplement, it is necessary to understand whether it harbors any toxicity. Since toxicological information on the topic is scarce, the present investigation was carried out to test whether rEP exhibits allergenic and/or toxic effects. In an oral acute toxicity test (OECD 425), Sprague Dawley (SD) rats were administered rEP dissolved in reverse osmosis water, yielding an LD_50_ > 5000 mg/kg (no observed animal death). The compound was therefore classified as non-toxic by oral administration. In an acute respiratory toxicity test (OECD 403), heads and noses of SD rats were exposed to liquid aerosol for 4 h (the highest concentration that could be administered without causing any animal death), and a lethal concentration (LC_50_) > 0.88 mg/L was obtained. The mass medium aerodynamics diameter (MMAD) of rEP aerosol particles was 8.18 μm and mass medium aerodynamics diameter (GSD) was 3.04, which meant that 25.90% could enter the airway (<4 μm) of a rat, and 58.06% (<10 μm) could be inhaled by humans. An ocular irritation test (OECD 405) with rEP powder was performed on New Zealand White (NZW) rabbits. Signs of irritation included conjunctival swelling and diffuse flushing 1 h after administration. The signs were less apparent after 24 h and disappeared after 72 h. The classification assigned to the powder was mild eye irritation. Skin sensitization was performed for a local lymphoproliferative test (OECD 442B) using BALB/c mice, with the highest soluble concentration of the rEP considered to be 100% test substance; formulations were diluted to 50% and 25%, and bromodeoxyuridine (BrdU) incorporation was used to measure the degree of lymphocyte proliferation. The stimulation indexes (SIs) were 1.06 (100%), 0.44 (50%), and 0.77 (25%), all of which were less than the cutoff value for a positive sensitization result (1.6). Negative response was also seen in the bacterial reverse mutation test (OECD 471), and no chromosomal effects on Chinese hamster ovary (CHO)-K1 cells were observed (OECD 487). Based on these six toxicity tests, rEP showed neither acute toxic effects in experimental animals nor mutagenicity. Thus, rEP can be considered safe for use in subsequent research on its application as a feed additive for poultry, cattle, or aquatic animals.

## 1. Introduction

The emergence of multidrug-resistant pathogens has necessitated the development of antibiotic alternatives to control deadly pathogens in humans and animals [1]. According to the World Health Organization (WHO), an alarming rise in death due to infections with multidrug-resistant pathogens is expected by the middle of this century [2]. The WHO has defined a category of drug-resistant pathogens (*Enterococcus faecium*, *Staphylococcus aureus*, *Klebsiella pneumoniae*, *Acinetobacter baumannii*, *Pseudomonas aeruginosa,* and *Enterobacter* spp.; abbreviated ESKAPE) that represent the most likely to cause a substantial increase in infectious cases around the globe [3]. In addition, several instances of polymicrobial infections with necrotizing fasciitis have been reported. In this condition, the microbial population feeds on the soft tissues in the infected individual, which may be fatal if left untreated for a sufficient duration [4]. Currently, there is an alarming rise in multidrug-resistant and pan-drug-resistant microbial species, along with a drying up of the drug discovery pipeline. Together, these developments have created an emergent need for potential antimicrobial therapeutics [5]. Antimicrobial peptides (AMPs) can be considered as a promising category of therapeutic agents due to their significant antimicrobial activity against drug-resistant pathogens [6].

AMPs are also known as host defense peptides, as they act against invading microbial pathogens [7]. Apart from their utility in innate immune response, anti-cancer and immunomodulatory activities are also reported for AMPs in various host organisms [8,9,10]. Defensins [11], cecropins [12], piscidins [13], and cathelicidins [14] are among the most widely-reported molecules of this class. These molecules are known to possess a net positive charge as well as amphipathicity. Based on tense structural features, AMPs electrostatically interact with the anionic bacterial membrane and cause membranolysis [15,16]. Unlike the mammalian membrane, the bacterial surface has a negative charge due to the presence of anionic lipids like 1-palmitoyl-2-oleoyl-sn-glycero-phosphocholine (POPC) and cardiolopins, as well as other anionic surface molecules, like lipoteichoic acid and lipopolysaccharide [17]. Despite the fact that AMPs possess significant therapeutic potential, their utility as drugs has not yet been established. Potential drawbacks, such as ion sensitivity and susceptibility to enzymatic degradation, can be overcome by introducing known molecular modifications that retain or enhance therapeutic utility [18].

In vitro and in vivo studies have been conducted on a vast array of potential AMPs, with the molecules showing significant activity, negligible toxicity, and lack of long-term allergenicity [8,19,20]. One group of potential therapeutic AMPs are the piscidins, which have been reported to possess strong antimicrobial activity against a variety of organisms [13]. Generally, AMPs are expressed by immune cells, i.e., mast cells [13]. The first ever report of AMPs was made about the genus *Morone* [13], and the molecules were found to be composed of 22 to 44 amino acids and exhibit an alpha-helical secondary structure. Since then, many other genera have been reported to express piscidin homologs. These molecules play important roles in maintaining immunological function in the host organisms, and they tend to exhibit alpha-helical conformations [21]. Studies have demonstrated their antimicrobial activity, and minimal cytotoxicity was evident in cellular studies [22,23]. In the current study, piscidin from *Epinephelus lanceolatus* was examined. This species is reported to possess 254 putative AMP genes, which may be important for maintaining the health of the fish [24]. We have described the isolation, subcloning of the expressed vector, and expression of the piscidin isolated from *Epinephelus lanceolatus* (rEP) in *Pichia pastoris* and identified the biological function of rEP in *Gallus domesticus* in our previous research [25]. rEP supplementation increased G. *g. domesticus* weight gain, feed efficiency, and IL-10 and IFN-γ production. Previously, we cloned and characterized five piscidin-like AMPs (named TP1–TP5) from *Oreochromis niloticus* [21]. The published results suggest that piscidins have broad-spectrum antibacterial, antifungal, antiviral, and antiparasitic activities. From our previous research results, we determined that *Epinephelus lanceolatus* piscidin (EP) exhibits high similarity to the highly-active TP3 and TP4 peptides from tilapia [21]. After investigating EP and other piscidins from fish by MIC methods, we chose the EP peptide as our target gene for expression in the yeast expression system. The EP experimental results suggested that EP possesses enhanced antimicrobial activity or growth inhibition activity in Gram-negative and Gram-positive bacteria [25].

Excessive use of antibiotics in feed supplements is a major concern, as this practice is correlated with the rise of drug-resistant bacterial species. The repetitive and improper dosing of antibiotics is one of the main reasons for the steep rise in multidrug-resistant pathogens. Many antibiotics are available over-the-counter, without stringent and ethical control, and are used by a large percentage of the population around the world [5]. Therefore, alternatives like AMPs can play a significant role in overcoming the current deficit of effective antibiotics [7]. AMP-containing feed supplementation may be a sound strategy to protect domestic animals, which might help prevent the excessive use of antibiotics [26]. Therefore, the commercial exploitation of AMPs as feed additives requires they first be examined in a stringent set of toxicological analyses. According to governmental regulatory bodies, there should be a thorough study of toxicity with model organisms, keeping safety aspects and health concerns as a priority during drug development [27]. This kind of toxicological analysis can provide evidence for the absence of toxic analytes/compounds that may be produced by unidentified modifications [27,28,29,30].

Toxicological analysis is an important part of the drug discovery process, as it helps to ensure the safe usage of newly-developed potential therapeutics. Tests like oral toxicity, inhalation toxicity, eye irritation, and skin sensitization are commonly conducted to assess the safety of test molecules [31]. Acute oral toxicity is generally evaluated by oral gavage of rats [32,33,34]. Similarly, acute inhalation toxicity studies are performed by exposing rat models to liquid aerosols for a given period of time [32,33]. Another established method of toxicological assessment is to instill the drug into the lower conjunctiva of the left eye of rabbit models and assess irritation [35]. Skin sensitization studies are also performed, where the test materials are applied to the skin behind the ears of BALB/c mice, and swelling (if it occurs) is taken as a measure of sensitization [36,37,38,39,40,41].

Toxicological studies on recombinant piscidin (rEP) expressed in *Pichia pastoris* are limited. Therefore, it is necessary to perform a panel of basic toxicity tests in accordance with preclinical guidelines of governmental regulatory bodies. In the present report, we evaluate the toxicity profile of rEP-expressing yeast pellets. In these tests, we saw that the test candidate did not cause significant oral toxicity, inhalation toxicity, eye irritation, skin sensitization, bacterial reverse mutation, or chromosomal effects. Thus, rEP may be considered safe for use in animal fodder as a supplement antibiotic substitute.

## 2. Results

### 2.1. Oral Toxicity in Rats

Oral intake of 5000 mg/kg body weight did not cause any mortality in test rats (Table 1). The body weight increases of treated rats on day 7 and day 14 were similar to controls (Table 2). No clinical signs or death of the treated rats was observed within the 14-day observation period (Table 1A). No significant gross lesions were found in any organs of rats surviving at day 14 of the experiment (Table 1). The acute oral LD_50_ for rEP was therefore greater than 5000 mg/kg body weight in female rats.

### 2.2. Inhalation Toxicity in Rats

rEP was formulated as six liters of liquid aerosol with 34.1% concentration, and Sprague Dawley (SD) rats were exposed for 4 h. A mean mass medium aerodynamics diameter (MMAD) of 8.18 µM and geometric standard deviation (GSD) of 3.04 were observed. Comparison with control animals revealed that these levels are safe and can be inhaled by the test animals without apparent harm. Some rats treated with rEP aerosol showed piloerection, chromodacryorrhea, hemorrhage of nose, and tachypnea for a short time, but these reactions disappeared after one day (Table 3). No mortality was observed, and weight gain of treated male rats was significantly different from blank controls on the 3rd and 14th day post-treatment (Table 4). Macroscopic lesions in the lungs of necropsy rats 14 days post-treatment had white protrusions and dark red spots (Table S1). Based on these experiments, the lethal concentration (LC_50_) of aerosol-administered rEP in rats was greater than 0.88 mg/L.

### 2.3. Eye Irritation in Rabbits

**Figure 1:**
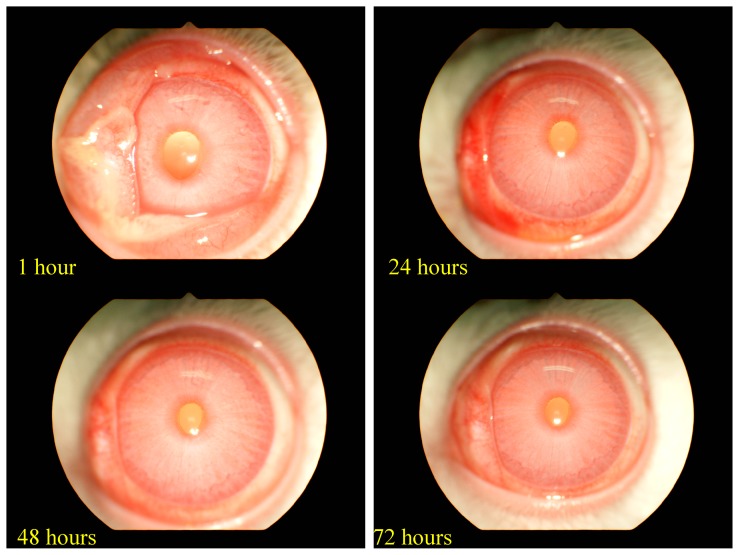
Recovery from rEP-mediated mild eye irritation after 24 h. Observation of eye irritation in an individual rabbit treated with rEP at 1, 24, 48, and 72 h post-treatment. Images were captured with a hand-held retinal camera.

An eye irritation test was conducted using NZW rabbits. No significant changes in body weight were observed between the time of rEP instillation and the end of the experiment. No obvious major clinical signs (except lacrimation) were observed during the test. After 0.1 g rEP was instilled into the left eye of each of three rabbits, none of the treated rabbits showed apparent turbidity in the cornea. The iris was clearly visible, and the cornea still reacted to the light, while the conjunctiva showed obvious swelling with lids about half-closed, having some discharge and diffuse, crimson color; individual vessels were not easily discernible. Observations of eye irritation for all three rabbits treated with rEP were made at 1, 24, 48, and 72 h post-treatment with a hand-held retinal camera. The clinical appearance of one representative treated eye is showed in Figure 1. After washing with reverse osmosis water at 24 h post-treatment, the minor signs were gradually lessened and all the signs disappeared by 72 h (Table 5).

### 2.4. Skin Sensitization in Mice

Application of the peptide pellet to skin did not cause significant changes in body weight. No obvious clinical signs, except rough coat, piloerection, alopecia, erythema, edema, and emaciation, were observed during the test (Table 6). The stimulation index (SI) was calculated for treatment groups (25%, 0.77; 50%, 0.44; and 100%, 1.06) and the positive-control group (1.69) (Table 7).

### 2.5. Bacterial Reverse Mutation Test

The results of the main test (including cytotoxicity test; conducted at 5000, 1667, 556, 185, 62, 21, and 7 μg/plate ± S9) are shown in Table 8 and Table 9. A confirmatory test (conducted at 5000, 2500, 1250, 625, and 313 μg/plate ± S9) was also conducted, and results are shown in Table 10 and Table 11. No treatment caused a significant elevation in revertants (2- or 3-fold more than negative controls) in any of the five tester strains. Whether addition of the S9 liver fraction or not had no apparent impact on cytotoxicity or precipitate formation. Therefore, we conclude that rEP exhibits a negative response in the bacterial reverse mutation test.

### 2.6. Micronucleus Test in Chinese hamster ovary (CHO)-K1 Cells

No treatments with rEP caused a significant increase in frequency of micronuclei compared to negative controls (0.5% DI water) in the micronucleus assay. In addition, a dose-response relationship was not observed (Table 12). The results showed that rEP had no observable effects on chromosomes (clastogenicity) of CHO-K1 cells.

## 3. Discussion

The serendipitous discovery of penicillin in the year 1928 was a monumental moment in the field of antimicrobial therapy [41]. However, the occurrence of resistance was reported as early as 1940, when an *E. coli* strain was found to inactivate penicillin by producing penicillinase enzyme [42]. Since then, numerous antibiotics have been discovered and applied. However, resistance to a large proportion has developed in microbial populations, resulting an increased global morbidity [5]. Apart from the clinically-used therapeutics, antibiotics have also been used as growth-promoting agents, for veterinary purposes, in aquaculture maintenance, and for other domestic uses. However, improper usage is thought to be the most important factor driving the evolution of antibiotic-resistant microbes [43].

The rise in global population has caused a sharp increase in the demand for food supply. Despite high production levels, a large quantity of food materials must be rejected due to microbial contamination. One prime reason for this persistent contamination is the recurrent rise of antibiotic-resistant microbial species. These drug-resistant species have limited the utility of the current spectrum of antibiotics. Factors like mutations, improper dosing, and non-compliance with recommendations are commonly responsible for the proliferation of drug-resistant species. Aquaculture is one example of a food industry that has been severely affected by problems with contamination. A wide variety of microbial species with acquired resistance to common antibiotics are found in aquaculture products. Hence, the constant monitoring of systems is crucial, as contamination can directly affect the general health of the entire culture, humans, and the environment [44]. To counter these issues, recombinant AMP-containing fodder has been evaluated in aquatic organisms [45]. AMPs are less prone to resistance than conventional antibiotics and they have been reported to kill multidrug-resistant microbial species [46]. Based on these previous promising results, more research on the use of AMPs as a fodder supplement in aquaculture is needed. Piscidin is a well-known AMP that is expressed in the mast cells of fish and exhibits potent antimicrobial activity [1,13]. Moreover, expression of AMPs, like piscidin, in yeast may serve as a suitable method to introduce the molecules as fodder supplements. Prior to application, it is necessary to investigate the possible toxicity and allergenic effects of AMP-expressing yeast pellets.

In this study, the main objective was to assess the toxicological effects of rEP-expressing yeast pellets before further evaluation of the product as a fodder supplement candidate in aquaculture and livestock industries. Oral toxicity, inhalation toxicity, eye irritation, and skin sensitization studies were conducted in vivo, and a bacterial reverse mutation test and micronucleus test in CHO-K1 cells were also conducted in accordance with OECD recommendations [47].

We performed eye irritation studies because the organ is located externally and it is highly susceptible to environmental factors. Moreover, the eye is extremely vascularized organ, so any long-term contact may allow molecules to enter systemic circulation. The eye itself is sensitive and prone to damage from chemical exposure [48]. We found that NZW rabbits instilled with rEP showed irritation within 1 h that lasted up to 72 h. Notably rEP treatment did not cause any long-term corneal opacity, conjunctival redness, or abnormality of the iris, and therefore, it can be considered as safe for eyes [49]. Apart from the ocular parameters, we also examined the body weight of the test animals. There was no notable difference in body weights prior to and after the treatment of test animals, indicating that the eye exposure route does not lead to overt systemic toxicity.

Pesticide poisoning through systemic or inhalation exposures is responsible for around 150,000 deaths annually [50]. Since inhaled agriculture and aquaculture materials, such as pesticides and herbicides, have been reported to cause harm to humans, it is necessary to assess the inhalation toxicity of any substance intended for use in aquaculture applications [51]. The inhalation experiments yielded an LC_50_ value for rEP that was greater than the maximal tested concentration of 0.88 mg/L, as no mortality was observed up to 14 days after inhalation exposure. Comparing the inhalation risk of rEP dust with the liquid aerosol inhalation toxicity study we conducted, the exposure to liquid aerosol allowed us to generate smaller particle than dust aerosol, and these smaller particles can easily access deeper regions of the lung. In the inhalation toxicity study, the liquid aerosol had an 8.18 μm MMAD and 3.04 GSD, meaning that about 25.90% of the aerosol can enter the airway of rats (<4μm), and about 58.06% of the aerosol could be inhaled by humans (<10μm). More than 20% of the test item could be inhaled by rats, and no death resulted. If inhalation exposure were performed with dust (bigger particle size than liquid aerosol), almost none of the chemical would enter the lungs and the acute inhalation toxicity should be reduced. In addition, the rEP-treated animals showed increased weight compared to 3 days after treatment. Since no mortality was observed, we can infer that the use of rEP in fodder should be safe.

The oral toxicity of pesticides is often the highest of any exposure route, especially for acetylcholinesterase inhibitors. Organophosphates, carbamates, and organochlorine are some such well-known pesticides [50,51,52]. Therefore, we evaluated the oral toxicity of rEP as a routine component of the toxicity analysis. In the oral toxicity study, we found that the acute oral LD_50_ of rEP was greater than 5 g/kg body weight in female rats. This result shows that even a very high concentration did not cause notable toxicity in test animals [33]. In addition, no mortality or significant variations in body weight were observed. Furthermore, clinical appearance and gross pathology were normal. These results allow us to conclude that the candidate lacks significant oral toxicity.

Murine ear is a common site to test allergenicity. In the hypersensitivity tests, animals were treated with 25 µL of 25%, 50%, and 100% rEP. After treatment, there was no notable change in the body weight over the 6 days of observation. Animals treated with the same doses were further examined, and erythema was observed at 25% rEP. Animals treated with the next two higher doses showed the symptoms of alopecia and erythema. However, only one individual from the 100% test group showed emaciation on the 6th day.

Since rEP may be considered for future fodder supplementation studies, a sensitization test is necessary to evaluate hypersensitivity and allergic reactions [43]. The skin sensitization study showed that the stimulation index (SI) values for 25%, 50%, and 100% rEP treatment groups were 0.77, 0.44, and 1.07, respectively, and the proliferation ratio of lymph node cells was less than 1.6 for all groups. These results indicate that rEP does not cause allergic or sensitization effects in mice. Taken together, the toxicity test results show that rEP is unlikely to cause significant eye, inhalation, oral, or contact toxicity, and it can be further evaluated as a fodder supplement in aquaculture.

## 4. Materials and Methods

### 4.1. Expression of Recombinant Piscidin in Fermenter Cultures

A yeast expression vector with the DNA sequence code for piscidin inserted after a methanol-inducible AOX promoter was cloned. After transformation into *Pichia pastoris*, a single colony was inoculated into 200 mL buffered glycerol-complex medium (BMGY) with PTM4 medium for 36 h at 28 °C, 200 rpm. The culture was transferred to a 5 L fermenter unit (Winpact, Major Science, Taoyuan, Taiwan) containing 3L commercial culture medium ((BMGY) and PTM4 medium) [36]. During fermentation, the temperature was maintained at 30 °C. The pH was adjusted to 6.0 with 14% ammonia and 0.1 N H_2_SO_4_. The concentration of dissolved oxygen was maintained above 20%. The fermenter culture was grown for 19 h, until the glycerol was completely consumed, followed by 50% w/v glycerol feeding for 360 min. Next, a 100% methanol feed was started and maintained for 24–96 h [37]. The cultures were then centrifuged at 6000 rpm for 30 min, and supernatants were spray-dried (YC-500, Shanghai Pilotech Instrument & Equipment Co, Ltd., Shanghai, China). Equal amounts of boiled lysate were separated on 12% polyacrylamide gels and then transferred to PVDF membranes. The membranes were incubated in blocking solution (0.1 M PBS, 5% nonfat milk, and 0.2% Tween-20) for 1 h at room temperature (RT) and then incubated in the same solution with the appropriate primary or secondary antibodies. rEP was detected by an anti-His-tag antibody (His-Tag Antibody (H-3): sc-8036, Santa Cruz Biotechnology, Inc., 10410 Finnell Street, Dallas, Texas 75220, U.S.A.) at a dilution of 1/6000. The second antibody used was an anti-mouse antibody at a dilution of 1/8000. Then, the membrane were washed three times with TBST buffer for 300 seconds each time. The membrane was incubated with chemiluminescent HRP substrate and detected by an imaging system (UVP, BioSpectrum 500, Analytik Jena US LLC (Formerly UVP LLC), Upland, CA, USA). Signal intensities were determined by densitometric analysis using the ImageJ program. Finally, the expression of rEP in fermentation cultures was confirmed by Western blotting, and rEP was quantified by comparison with known concentrations of synthetic piscidin peptide [25]. The rEP concentration was 262.9 µg/g (Figure S1).

### 4.2. Toxicology Studies

Pellets of yeast expressing rEP were batch produced in a 100-liter fermenter system and analyzed by the Taiwan Agricultural Chemicals and Toxic Substances Research Institute (TACTRI), Taichung, Taiwan, for acute oral toxicity, acute inhalation toxicity, acute eye irritation, skin sensitization, bacterial reverse mutation testing, or chromosomal effects in CHO-K1 cells. These experimental methods for each test are detailed below. All of the studies were performed in accordance with the guidelines from OECD, principles of GLP, and US EPA GLP regulations in the Insecticide, Bactericide, and Rodenticide Act (FIFRA). The animal use protocol was reviewed and approved (19-TACTRI-IACUC-6, 19-TACTRI-IACUC-15, No.19-TACTRI-IACUC-11, No.19-TACTRI-IACUC-14) by the Institutional Animal Care and Use Committee (IACUC) of TACTRI. Sprague-Dawley (SD) rats were purchased from BioLASCO (Taipei, Taiwan, ROC), and the studies were performed on rats under the age of 10–11 weeks (young adult). New Zealand White (NZW) rabbits were purchased from the Livestock Research Institute, COA, EY (Tainan, Taiwan, ROC), and the study was performed on animals under the age of 17 weeks (young adult). BALB/c mice were purchased from the National Laboratory Animal Center (Taipei, Taiwan, ROC), and the studies were performed on animals under 10 weeks of age (young adult). During the experiments, rats (one to three per cage) or mice (five per cage) were housed in polycarbonate (PC) cages with stainless covers, and rabbits were housed individually in stainless steel cages. All animals were housed under controlled environmental conditions, including a temperature of 22 ± 3 °C, a relative humidity of 55 ± 15%, a ventilation more than 15 air exchanges per hour with HEPA filtered air, and a 12 h light/dark cycle (light period, 06:00–18:00). Drinking water and pelleted rodent diet were available ad libitum. After a quarantine period, animals in good health, based on clinical observation and body weight, were selected for experiments.

### 4.3. Acute Oral Toxicity Study

The procedures were consistent with OECD test guideline 425 (2008). Ten-week-old female SD rats were administered a dose of 5000 mg/kg body weight of rEP by gavage and observed for clinical signs, weight gain, mortality, and gross lesions for 14 days.

### 4.4. Acute Inhalation Toxicity Study

The procedures were consistent with OECD test guideline 403 (2009). A pretest was performed with a head-nose-only exposure system using rEP liquid aerosol for 4 h on a group of four SD rats with equal numbers of males and females; no death was observed in the pretest. Five male and five female SD rats at 11 weeks of age were used for the main inhalation toxicity test. The rEP powder was autoclaved before administration. The temperature, relative humidity, and concentration of liquid aerosol in the inhalation chamber were analyzed. Before and after weights of filter paper or metal collection sheets in the cascade impactor were analyzed for particle size distribution. After exposure the rats were observed for 14 days and clinical signs were recorded, in addition to body weight and survival rate. After 14 days, all rats were sacrificed and necropsies were performed.

### 4.5. Acute Eye Irritation Study

The procedures were consistent with OECD test guideline 405 (2017). Acute eye irritation was assessed at TACTRI on the eye of NZW rabbits. rEP (0.1 g) was instilled into the lower conjunctiva sac of the animal [39]. Seventeen-week-old nulliparous and non-pregnant animals were acclimatized for one week and used in the study. The left eyes of three rabbits were used for the test, and one day prior to testing, both eyes were examined to ensure that there was no preexisting corneal damage. In accordance with OECD guidelines, no reference was used in this study. An aliquot of 0.1 g rEP was placed into the lower conjunctiva sac of the left eye in each treatment rabbit. Proparacaine hydrochloride (0.5%) and buprenorphine (0.01 mg/kg) were applied as topical anesthetics and systemic analgesics. Examination of the eyes was performed at 1, 24, 48, and 72 h after instillation. Fluorescein dye (2% Fluorescein sodium, Merck KGaA, Darmstadt, Germany) and a hand-held retinal camera (GENESIS, Kowa, Tokyo, Japan) were used to confirm corneal ulceration in treated eyes. If any signs of irritation were noted at 72 h, extra observations were made at 7, 14, and 21 days. All signs of irritation were noted to thoroughly evaluate the irritation of the cornea (degree/area of opacity), iris (damage value), and conjunctiva (redness and chemosis) (Figure S2). Cornea visibility and reactivity to light, swelling of lids, visibility of eye vessels, and discharge were also examined to assess eye irritation. Day of treatment was day 1, and routine clinical signs (increased motor activity, rough coat, reduced motor activity, piloerection, flaccid, tachypnea, stiffness, dyspnea, tremor, nostril discharge, ataxia, diarrhea, paralysis, fasciculation, exophthalmos, paleness, lacrimation, erythema, salivation, and edema) were recorded on a daily basis [40].

### 4.6. Skin Sensitization Study (Local Lymph Node Assay)

The procedures were consistent with OECD test guideline 442B (2018). The test method had the advantages of being quantitative, having a short test period (about 1 week), and reducing the pain and use of animals (more than 50% compared with OECD 406). LLNA- bromodeoxyuridine (BrdU) was used to test the potential of skin sensitization of rEP, and to analyze whether the test compound stimulated an allergic reaction [53]. Twenty-five microliters of 25%, 50%, and 100% rEP solution was applied to the back of Balb/C mice ears for three consecutive days. Six days post-treatment, the mice were sacrificed and proliferation of lymph node cells was analyzed by bromodeoxyuridine (BrdU) incorporation into cells using an ELISA read by measuring absorbance at wavelengths 370 and 492 nm [41]. The stimulation index (SI) was derived by dividing the mean BrdU labelling index/mouse within each treatment group and positive control group by the mean BrdU labelling index for the solvent control group. When the proliferation ratio of lymph node cells reached 1.6 times or above (SI ≥ 1.6), the test item was considered to have a potential positive sensitization effect.

### 4.7. Bacterial Reverse Mutation Test

The procedures were consistent with OECD test guideline 471 (1997) and USEPA OCSPP (formerly OPPTS) harmonized test guidelines 870.5100 (1998) without any modification. Mutagenicity of rEP (262.9 µg/g) was examined in the bacterial reverse mutation test with *Salmonella typhimurium* strains TA98, TA100, TA1535, TA1537, and TA102 by the plate incorporation method. Rat liver S9 enzymes were used to mimic a mammalian metabolic activation system. rEP, a light yellow powder, was dissolved in deionized (DI) water to make working solutions. The assay was performed with two independent replications (main test and confirmatory test) using indicated concentrations and included blank, negative, and positive controls in triplicate.

### 4.8. Micronucleus Test in CHO-K1 Cell

The procedures were consistent with OECD test guideline 487 (2016). The rEP (concentration 262.9 µg/g) was evaluated by cytokinesis-block micronucleus (CBMN) assay with Chinese hamster ovary (CHO-K1) cells. Rat liver S9 enzymes were used to mimic a mammalian metabolic activation system of biotransformation. Concentrations of rEP tested in the micronucleus assay were selected based on the results of the cytotoxicity range finder and solubility test. The concentrations selected for evaluation were 125, 250, 500, 1000, and 2000 μg/mL for short-term treatment (3 + 20) hours with and without S9 mix and long-term treatment (24 + 0) hours without S9 mix.

## 5. Conclusions

Aquaculture systems are water bodies that provide a rich environment for the growth of microorganisms. Of late, many microbes have acquired resistance to a wide spectrum of commercial antibiotics. Hence, the rise of such super bugs has resulted in major problems for food production. Infections in food production are not only detrimental to environmental health, but also cause huge losses in the global economy.

The current study was performed to evaluate the safety of a promising formulation for piscidin, which can be used as a fodder supplement in aquaculture. The peptide has shown bactericidal activity [13], and here we assessed a routine panel of toxicological parameters, as per OECD guidelines. The peptide piscidin showed negligible toxicity in all the tests. Hence, this formulation may be considered for further development as a potential supplement for aquaculture fodder.

## Figures and Tables

**Table 1:** Oral administration of 5000 mg/kg recombinant piscidin (rEP) did not affect examined rats. Survival, mortality, clinical signs, and gross pathology after oral dosing of rats.

Dose Level (mg/kg)	Dosing Volume (mL/rat)	Survival	Mortality (%)	Observation Period (Day)	Clinical Signs	Gross Pathology Finding *
5000 (3/3) **	2.4	Survival (3/3) **	0	14	None (3/3) **	None (3/3) **

* The gross pathology examination was performed on anus, heart, lung, stomach, liver, pancreas, digestive tract, kidney, thymus, and spleen; ** number of animals/total number of examined animals.

**Table 2:** Oral administration of 5000 mg/kg recombinant piscidin (rEP) did not affect examined rats. Weekly body weights and body weight gain of orally-treated rats.

Dose Level (mg/kg)	Body Weight (g) (Mean ± SD)			Body Weight Gain * (g) (Mean ± SD)		Body Weight Gain ** (%) (Mean ± SD)	
	Day 0	Day 7	Day 14	Day 7	Day 14	Day 7	Day 14
5000	240.7 ± 3.2	257.6 ± 8.3	272.6 ± 9.9	16.9 ± 5.2	31.9 ± 6.8	7.0 ± 2.1	13.2 ± 2.7

* Body weight (BW) gain (g) on day N = (day N − day 0) BW; ** body weight (BW) gain (%) on day N = [(day N − day 0)/day0] BW × 100%. SD, Standard deviation.

**Table 3:** Clinical signs in rats exposed to 0.88 mg/L liquid aerosol rEP.

Time	Clinical Signs *	
	Male	Female
1 h	tachypnea (5/5)	tachypnea (5/5)
2 h	tachypnea (5/5), abdominal breathing (5/5)	tachypnea (5/5), abdominal breathing (5/5)
4 h	piloerection (5/5), chromodacryorrhea (4/5), hemorrhage of nose (1/5), tachypnea (5/5)	piloerection (5/5), chromodacryorrhea (5/5), tachypnea (5/5)
1 to 14 Days	None (5/5)	None (5/5)

* Number of animals/total number of animals; * observation items: overall normal or not; death; appearance of coat skin and mucus; appearance of mouth, eyes, and nose; behavior, movement, and posture; the reflex system; and the respiratory and digestive systems.

**Table 4:** Weight of rats treated with rEP by inhalation. Sprague Dawley (SD) rats were exposed to rEP liquid aerosol inhalation chamber for 4 h. After 1, 3, 7, and 14 days, weight gain percent was calculated.

Sex		Group	Concentration of rEP (mg/L)	Weight Gain (%) ^1^	
				Day 1 ^2^	Day 3 ^2^
Male		Blank Control	0	−0.4 ± 2.5	−6.0 ± 0.9
		Treated group	0.880	1.6 ± 0.9	−1.7 ± 2.4 *
Female		Blank Control	0	−0.5 ± 1.5	0.1 ± 2.8
		Treated group	0.880	0.1 ± 2.1	−1.6 ± 3.9
Combined		Blank Control	0	−0.5 ± 1.9	−2.9 ± 3.8
		Treated group	0.880	0.8 ± 1.7	−1.7 ± 3.1
**Sex**		**Group**	**Concentration of rEP (mg/L)**	**Weight Gain (%) ^1^**	
				**Day 7 ^2^**	**Day 14 ^2^**
Male		Blank Control	0	3.5 ± 2.2	7.8 ± 3.5
		Treated group	0.880	0.9 ± 2.2	3.0 ± 2.1 *
Female		Blank Control	0	1.9 ± 3.1	0.5 ± 6.0
		Treated group	0.880	3.3 ± 2.2	4.4 ± 5.6
Combined		Blank Control	0	2.7 ± 2.6	4.1 ± 6.0
		Treated group	0.880	2.1 ± 2.5	3.7 ± 4.1

^1^ Data for each sex were derived from 5 treated animals and the combined value represents 10 treated animals; * indicates significant difference (p < 0.05) from control group by Student’s t test; ^2^ weight gain (%) on day n = (body weight of day n – body weight of day 0)/(body weight of day 0) × 100%.

**Table 5:** Ocular score of rabbits treated with rEP. The left eyes of NZW rabbits were instilled with 0.1 g rEP in the lower conjunctiva sac, and the mean irritation score (Supplementary Figure S2) was calculated at 1, 24, 48, and 72 h post-treatment. N = 3.

Hour	Irritant Scoring				
	Cornea		Iris	Conjunctiva	
	Degree	Area	Damage	Redness	Chemosis
1	0	0	1	1.67	2.67
24	0	0	1	1.67	2
48	0	0	0	1	1
72	0	0	0	0	0

**Table 6:** Clinical signs and body weight of mice after treatment of ears with rEP. Twenty-five microliters of 25%, 50%, or 100% rEP solution was applied to the back of Balb/C mice ears for three consecutive days. Sterile water, AOO *, and 2% 2,4-dinitro-1-chlorobenzene (DNCB) were used as controls. Body weight before treatment and six days after treatment (before sacrifice) are shown.

	Groups					
	Sterile Water	AOO *	2% DNCB	25% rEP	50% rEP	100% rEP
Time (days) ^1^	Clinical signs **					
1	None (5/5)	Rough coat (5/5)	Rough coat (5/5), Piloerection (5/5), Erythema (5/5)	None (5/5)	Erythema (4/5)	Erythema (4/5)
2	None (5/5)	Rough coat (5/5), Erythema (5/5)	Rough coat (5/5), Piloerection (5/5), Erythema (5/5), Edema (5/5)	Erythema (3/5)	Erythema (5/5)	Alopecia (3/5), Erythema (5/5)
3	None (5/5)	Rough coat (5/5), Erythema (5/5)	Rough coat (5/5), Piloerection (5/5), Erythema (5/5), Edema (5/5)	Erythema (5/5)	Alopecia (3/5), Erythema (5/5)	Rough coat (1/5), Alopecia (5/5), Erythema (5/5)
4	None (5/5)	Rough coat (5/5), Erythema (5/5)	Rough coat (5/5), Piloerection (5/5), Erythema (5/5), Edema (5/5)	Erythema (5/5)	Alopecia (5/5), Erythema (5/5)	Alopecia (5/5), Erythema (5/5)
5	None (5/5)	Rough coat (1/5), Erythema (5/5)	Rough coat (5/5), Piloerection (5/5), Erythema (5/5), Edema (5/5)	Erythema (5/5)	Alopecia (5/5), Erythema (5/5)	Alopecia (5/5), Erythema (5/5)
6	None (5/5)	Erythema (5/5)	rough coat (5/5), piloerection (5/5), erythema (5/5), edema (5/5)	Erythema (2/5)	Alopecia (5/5), Erythema (2/5)	Alopecia (5/5), Erythema (5/5), Emaciation (1/5)
Time (treatment)	Body weight (g) (mean ± SD)					
Before dosing	21.4 ± 1.2	22.0 ± 0.5	21.5 ± 1.0	22.0 ± 0.7	21.7 ± 0.5	21.8 ± 1.3
After dosing (before sacrifice)	21.4 ± 1.1	22.4 ± 0.9	22.0 ± 0.8	22.5 ± 0.6	21.9 ± 0.6	21.2 ± 1.6

* AOO: acetone: olive oil (4:1 *v*/*v*); ** (number of animals/total number of examined animals); ^1^ day 1 was defined as the day of treatment.

**Table 7:** rEP did not cause sensitization of mice in a bromodeoxyuridine (BrDU) assay. Lymph nodes cells were labeled with BrdU, and the BrdU-labeling index was estimated by measuring absorbance at 370 and 492 nm in an ELISA reader. The stimulation index (SI) value was calculated as the ratio of control and treatment groups. SI value less than 1.6 means the test item did not cause skin sensitization.

Group	Treatment BrdU Labelling Index ÷ Solvent Control BrdU Labelling Index = SI Value				
**2% DNCB**	**0.405**	**÷**	**0.239**	=	1.69
25% rEP	0.179	÷	0.231	=	0.77
50% rEP	0.102	÷	0.231	=	0.44
100% rEP	0.244	÷	0.231	=	1.06

**Table 8:** Revertants of *Salmonella typhimurium* TA strains treated with rEP peptide (marine peptide) in the absence of S9 mix (main test including cytotoxicity) ^(a)^.

Treatment	S9	TA98	TA100	TA1535	TA1537	TA102
	±	Mean ± SD	Mean ± SD	Mean ± SD	Mean ± SD	Mean ± SD
BK ^(b)^	-	23.0 ± 1.7	119.7 ± 17.7	8.7 ± 1.5	8.0 ± 1.0	200.0 ± 13.1
NC ^(c)^	-	24.3 ± 1.5	110.0 ± 9.6	9.7 ± 1.5	8.7 ± 1.2	205.3 ± 16.8
PC ^(d)^	-	440.7 ± 40.5 **(18.1)	>2000 ^(e)^**(18.2)	1425.3 ± 113.2 **(147.4)	>2000 ^(e)^**(230.8)	>2000 ^(e)^**(9.7)
Marine peptide (μg/plate)						
7	-	21.0 ± 1.0(0.9)	118.0 ± 19.3(1.1)	10.7 ± 1.2(1.1)	9.7 ± 2.3(1.1)	205.0 ± 22.6(1.0)
21	-	21.7 ± 3.8(0.9)	120.3 ± 18.6(1.1)	8.7 ± 2.1(0.9)	9.3 ± 1.5(1.1)	193.3 ± 14.6(0.9)
62	-	24.0 ± 0.0(1.0)	125.0 ± 13.5(1.1)	10.0 ± 2.0(1.0)	9.0 ± 1.7(1.0)	192.3 ± 32.9(0.9)
185	-	26.0 ± 5.0(1.1)	121.3 ± 4.7(1.1)	11.3 ± 3.1(1.2)	10.3 ± 2.3(1.2)	186.7 ± 26.8(0.9)
556	-	24.3 ± 2.1(1.0)	126.7 ± 5.5(1.2)	11.7 ± 1.5(1.2)	11.3 ± 3.1(1.3)	195.0 ± 16.0(0.9)
1667	-	28.7 ± 2.3(1.2)	116.0 ± 19.0(1.1)	12.0 ± 3.6(1.2)	10.0 ± 3.0(1.2)	193.3 ± 9.6(0.9)
5000	-	27.7 ± 1.2(1.1)	135.0 ± 8.2(1.2)	12.0 ± 2.0(1.2)	6.0 ± 1.7(0.7)	196.3 ± 20.0(1.0)
Dose response ^(f)^	0.845 **		0.283	0.602 *	0.115	0.105

^(a)^ Data represent means of three replicates. Numbers in parentheses are fold-induction compared with negative control (NC); ^(b)^ blank control (BK); ^(c)^ negative control (NC): 100 μL/plate deionized water (DI water); and ^(d)^ positive control (PC): in assay without liver S9: 0.5 μg/plate 4-nitroquinoline-N-oxide (TA98 and TA100), 5 μg/plate sodium azide (TA1535), 125 μg/plate 9-aminoacridine (TA1537), and 0.5 μg/plate mitomycin C (TA102). ** *p* < 0.01 compared to NC (Student’s t test, one-tail); ^(e)^ Data were analyzed with 2000 colony numbers/plate; and ^(**f**)^ dose response: correlation was analyzed for doses without significant cytotoxicity and revertants per plate. * *p* < 0.05. ** *p* < 0.0.1.

**Table 9:** Revertants of *Salmonella typhimurium* TA strains treated with rEP peptide in the presence of S9 mix (main test including cytotoxicity) ^(a)^.

Treatment	S9	TA98	TA100	TA1535	TA1537	TA102
	±	Mean ± SD	Mean ± SD	Mean ± SD	Mean ± SD	Mean ± SD
BK ^(b)^	+	24.0 ± 1.0	126.7 ± 10.0	9.7 ± 0.6	7.3 ± 0.6	227.0 ± 18.1
NC ^(c)^	+	26.0 ± 3.6	133.7 ± 12.1	10.7 ± 1.5	9.0 ± 1.7	214.3 ± 4.7
PC ^(d)^	+	>2000 ^(e)^ **(76.9)	1812.7 ± 106.7 **(13.6)	227.7 ± 23.9 **(21.3)	117.0 ± 24.9 **(13.0)	1228.0 ± 200.0 **(5.7)
Marine peptide (μg/plate)						
7	+	26.3 ± 3.1(1.0)	120.3 ± 17.1(0.9)	11.0 ± 1.7(1.0)	9.3 ± 2.3(1.0)	210.7 ± 12.5(1.0)
21	+	26.7 ± 3.5(1.0)	116.3 ± 20.0(0.9)	10.0 ± 1.0(0.9)	8.7 ± 0.6(1.0)	215.7 ± 18.0(1.0)
62	+	23.3 ± 3.2(0.9)	118.3 ± 21.6(0.9)	9.3 ± 1.5(0.9)	8.3 ± 2.3(0.9)	202.3 ± 9.7(0.9)
185	+	25.7 ± 3.2(1.0)	128.3 ± 18.2(1.0)	11.7 ± 2.5(1.1)	7.7 ± 1.5(0.9)	203.0 ± 24.6(0.9)
556	+	23.0 ± 3.0(0.9)	118.0 ± 9.2(0.9)	10.3 ± 2.5(1.0)	9.3 ± 2.5(1.0)	202.0 ± 24.6(0.9)
1667	+	25.7 ± 3.2(1.0)	115.0 ± 10.4(0.9)	13.7 ± 1.5(1.3)	8.0 ± 2.0(0.9)	211.3 ± 16.3(1.0)
5000	+	25.7 ± 2.9(1.0)	138.3 ± 11.0(1.0)	9.7 ± 1.2(0.9)	7.7 ± 1.5(0.9)	202.3 ± 24.3(0.9)
Dose response ^(f)^		0.054	0.222	0.050	0.331	0.215

^(a)^ Data represent means of three replicates. Numbers in parentheses are fold-induction compared with NC; ^(b)^ blank control (BK); ^(c)^ negative control (NC): 100 μL/plate deionized water (DI water); and ^(d)^ positive control (PC): in assay with liver S9: 5 μg/plate 2-aminofluorene (TA98), 10 μg/plate 2-aminofluorene (TA100), 5 μg/plate 2-aminoanthracene (TA 1535), 20 μg/plate 2-aminofluorene (TA1537), and 30 μg/plate danthron (TA102). ** *p* < 0.01 compared to NC (Student’s t test, one-tail); ^(e)^ Data were analyzed with 2000 colony numbers/plate; and ^(f)^ dose response: correlation was analyzed for doses without significant cytotoxicity and revertants per plate.

**Table 10:** Revertants of *Salmonella typhimurium* TA strains treated with rEP peptide in the absence of S9 mix (confirmation test) ^(a)^.

Treatment	S9	TA98	TA100	TA1535	TA1537	TA102
	±	Mean ± SD	Mean ± SD	Mean ± SD	Mean ± SD	Mean ± SD
BK ^(b)^	-	23.0 ± 2.6	113.0 ± 14.4	11.0 ± 1.0	9.0 ± 2.6	200.7 ± 9.0
NC ^(c)^	-	25.7 ± 3.5	105.7 ± 7.2	13.7 ± 2.5	8.3 ± 0.6	202.0 ± 27.2
PC ^(d)^	-	445.0 ± 35.8 **(17.3)	>2000 ^(e)^**(18.9)	1488.0±150.7 **(108.9)	>2000 ^(e)^**(240.0)	>2000 ^(e)^**(9.9)
Marine peptide						
(μg/plate)						
313	-	24.7 ± 0.6(1.0)	112.3 ± 4.9(1.1)	12.3 ± 3.1(0.9)	7.7 ± 1.5(0.9)	203.0 ± 8.9(1.0)
625	-	26.0 ± 1.0(1.0)	100.0 ± 7.2(0.9)	9.7 ± 1.2(0.7)	9.3 ± 2.1(1.1)	195.7 ± 8.5(1.0)
1250	-	25.7 ± 6.1(1.0)	112.0 ± 9.5(1.1)	9.0 ± 0.0(0.7)	7.7 ± 0.6(0.9)	197.3 ± 25.9(1.0)
2500	-	26.7 ± 3.1(1.0)	136.7 ± 24.7(1.3)	10.3 ± 2.5(0.8)	11.0 ± 2.6(1.3)	225.7 ± 30.7(1.1)
5000	-	24.7 ± 0.6(1.0)	126.3 ± 28.5(1.2)	10.7 ± 2.1(0.8)	8.3 ± 2.1(1.0)	216.0 ± 30.8(1.1)
Dose response ^(f)^		0.015	0.517	0.113	0.114	0.471

^(a)^ Data represent means of three replicates. Numbers in parentheses are fold-induction compared with NC; ^(b)^ blank control (BK); ^(c)^ negative control (NC): 100 μL/plate deionized water (DI water); and ^(d)^ positive control (PC): in assay without liver S9: 0.5 μg/plate 4-nitroquinoline-N-oxide (TA98 and TA100), 5 μg/plate sodium azide (TA1535), 125 μg/plate 9-aminoacridine (TA1537), and 0.5 μg/plate mitomycin C (TA102). ** *p* < 0.01 compared to NC (Student’s t test, one-tail); ^(e)^ Data were analyzed with 2000 colony numbers/plate; and ^(f)^ dose response: correlation was analyzed for doses without significant cytotoxicity and revertants per plate. * *p* < 0.05.

**Table 11:** Revertants of *Salmonella typhimurium* TA strains treated with rEP peptide in the presence of S9 mix (confirmation test) ^(a)^.

Treatment	S9	TA98	TA100	TA1535	TA1537	TA102
	±	Mean ± SD	Mean ± SD	Mean ± SD	Mean ± SD	Mean ± SD
BK ^(b)^	+	22.7 ± 2.3	109.0 ± 4.0	10.7 ± 2.1	7.3 ± 1.2	209.7 ± 18.1
NC ^(c)^	+	25.3 ± 2.5	105.3 ± 1.5	11.7 ± 3.2	10.3 ± 0.6	208.3 ± 4.7
PC ^(d)^	+	>2000 ^(e)^**(78.9)	961.3 ± 82.8 **(9.1)	167.0 ± 13.0 **(14.3)	100.7 ± 10.3 **(9.7)	1087.7 ± 160.0 **(5.2)
Marine peptide (μg/plate)						
313	+	26.3 ± 6.1(1.0)	97.7 ± 2.3(0.9)	8.7 ± 2.1(0.7)	10.3 ± 2.9(1.0)	209.7 ± 7.6(1.0)
625	+	30.0 ± 2.6(1.2)	97.3 ± 11.0(0.9)	13.7 ± 2.1(1.2)	11.3 ± 2.5(1.1)	185.7 ± 20.6(0.9)
1250	+	19.7 ± 3.1(0.8)	102.0 ± 4.0(1.0)	13.3 ± 2.9(1.1)	7.3 ± 0.6(0.7)	187.7 ± 14.2(0.9)
2500	+	23.0 ± 2.0(0.9)	99.3 ± 7.8(0.9)	12.0 ± 2.0(1.0)	8.3 ± 2.1(0.8)	199.0 ± 12.5(1.0)
5000	+	29.0 ± 5.3(1.1)	107.7 ± 14.6(1.0)	12.3 ± 2.5(1.1)	10.7 ± 1.2(1.0)	185.7 ± 15.6(0.9)
Dose response ^(f)^		0.004	0.667	0.204	0.048	0.268

^(a)^ Data represent means of three replicates. Numbers in parentheses are fold-induction compared with NC; ^(b)^ blank control (BK); ^(c)^ negative control (NC): 100 μL/plate deionized water (DI water); ^(d)^ positive control (PC): in assay with liver S9: 5 μg/plate 2-aminofluorene (TA98), 10 μg/plate 2-aminofluorene (TA100), 5 μg/plate 2-aminoanthracene (TA 1535), 20 μg/plate 2-aminofluorene (TA1537), and 30 μg/plate danthron (TA102). ** *p* < 0.01 compared to NC (Student’s t test, one-tail); ^(e)^ Data were analyzed with 2000 colony numbers/plate; and ^(f)^ dose response: correlation was analyzed for doses without significant cytotoxicity and revertants per plate.

**Table 12:** Micronucleus analysis.

Exposure Time	(3 + 20) h-S9	(3 + 20) h + S9	(24 + 0) h-S9
Treatment	Micronucleus Assay		
(μg/mL)	Frequency of Micronuclei (%) ^(a)^		
BK ^(b)^	4.10	3.25	4.85
NC ^(c)^	3.75	3.85	4.75
125	4.55	3.95	4.60
250	3.95	4.00	3.95
500	3.55	3.25	5.20
1000	3.80	3.15	4.85
2000	3.90	3.75	5.55
PC ^(d)^	9.60 **	7.20 **	11.40 **
Dose response (*p* value) ^(e)^	0.601	0.299	0.134

^(a)^ Frequency of micronuclei (%). Data were analyzed by a chi-squared test with Yate’s correction (GraphPad 5), ** *p* < 0.01 compared to NC. Micronuclei were scored in at least 2000 cells per treatment (at least 1000 cells per culture; two cultures per concentration); ^(b)^ blank control (BK); ^(c)^ negative control (NC): 0.5% DI water; ^(d)^ positive control (PC): 0.25 μg/mg mitomycin C (−S9); 10 μg/mg cyclophosphamide (+S9); and ^(e)^ dose response (trend test) (GraphPad 5).

## Supplementary Materials

The following are available online at https://www.mdpi.com/1660-3397/18/4/206/s1. Figure S1: Quantitative analysis of synthesized peptides and rEP was accomplished by Western blotting. The detailed experimental methods are described in Materials and Methods (Section 4.1). The upper left panel shows the EP synthesis (50, 40, and 20 µg/mL) used to calculate the concentrations and standard concentrations. The 50 indicates that we loaded 50 ng of synthetic peptide in the well. After Western blotting, the membrane was incubated with chemiluminescent HRP substrate and detected by an imaging system (UVP, BioSpectrum 500, Analytik Jena US LLC (Formerly UVP LLC), Upland CA, USA). Signal intensities were determined by densitometric analysis using the ImageJ program. The upper right panel shows the calculated rEP amount from the methanol induction for 72 h. The bottom left panel shows the quantitation of rEP in the Western blotting experiment. M is the protein marker; P is the synthetic peptide (40 µg/mL) (in the well containing 40 ng of synthetic peptide); 0 indicates not treated with methanol; 3 indicates treatment with methanol for 72 h; and V is the expression vector only without any EP gene insertion as a control, Figure S2: Criteria for the evaluation of eye irritation. Lower conjunctiva sac of the left eye in NZW rabbits were treated with 0.1 g of rEP and irritancy description scores were recorded 1, 24, 48, and 72 hours after treatment. (A1) Degree of opacity; (A2) area of opacity in cornea; (B) iris damage value; (C1) redness; and (C2) chemosis in conjunctiva. Table S1: Macroscopic findings in treated rats at 14 days after inhalation of liquid aerosol rEP. Sprague Dawley (SD) rats were exposed to of 0.88 mg/L rEP liquid aerosol in an inhalation chamber for 4 h. After 14 days, the rats were examined for macroscopic abnormalities.
