# Supplementary material for: Lack of Acute Toxicity and Mutagenicity from Recombinant Epinephelus lanceolatus Piscidin Expressed in Pichia pastoris
Source: Mar Drugs. 2020 Apr 11;18(4):206. doi: 10.3390/md18040206 (PMC7230367; doi:10.3390/md18040206)
Supplement: Supplementary file 1 [file marinedrugs-18-00206-s001.pdf]

Supplementary

Table 1. Macroscopic findings in treated rats 14 days after liquid aerosol inhalation of Piscidin pellets. Sprague Dawley (SD) rats were exposed to of 0.88 mg/L Piscidin pellet liquid aerosol in an inhalation chamber for 4 h. After 14 days, the rats were examined for macroscopic abnormalities.

Group	ID of animal	Survival/Death	Macroscopic finding
**Blank control without exposure**	B01	Survival	None
	B05	Survival	None
**Treated group (0.88 mg/L)**	101	Survival	Multiple local dark red spots and protrusion on left lobe of dorsal lung. Left lobe of ventral lung had a white protrusion.
	102	Survival	Left lobe of dorsal lung had a dark red spot.
	103	Survival	Left lobe of dorsal lung had a dark red plaque. Multiple local white protrusions and dark red plaques on ventral lung.
	104	Survival	Multiple local dark red spots on left lobe of ventral lung.
	105	Survival	Multiple local white protrusions on left lobe of dorsal and ventral lung.
	106	Survival	Multiple local white protrusions on dorsal lung
	107	Survival	Multiple local white protrusions on dorsal lung. Right caudal lobe of dorsal lung had a dark red spot close to the bronchi
	108	Survival	Right caudal lobe of ventral lung had a white protrusion
	109	Survival	Multiple local dark red plaques and white protrusions on dorsal lung. One white protrusion and multiple local dark red plaques/spots on ventral lung.
	110	Survival	Multiple local dark red plaques and white protrusions on left lobe of dorsal lung. Multiple local white protrusions on right caudal lobe of ventral lung.

Items observed: Respiratory system, heart, liver, pancreas, kidneys, adrenal gland, thymus, spleen, and testes or ovaries

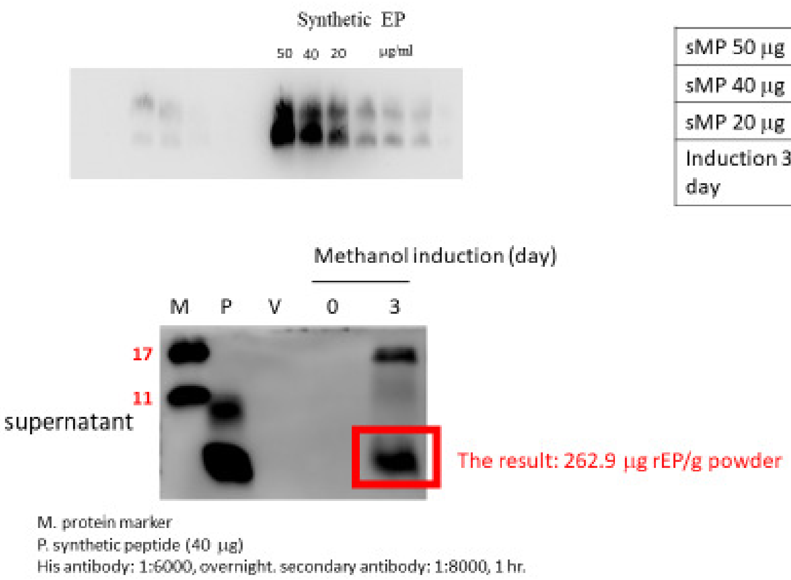

ImageJ

sMP 50 μg	1617.96	
sMP 40 μg	1113.77	
sMP 20 μg	235.97	
Induction 3 day	514.77	26.29 μg

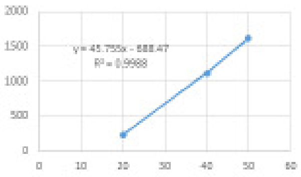

Supplementary figure 1

Quantitative analysis of synthesized peptides and rEP was accomplished by Western blotting. The detailed experimental methods are described in “5. Materials and Methods (5.1. Expression of recombinant piscidin in fermenter cultures)”. The upper left panel shows the EP synthesis (50, 40, 20 μg/ml) to calculate the concentrations and standard concentrations. The 50 indicates that we are loading 50 ng of synthetic peptide in the well. After Western blotting, the membrane was incubated with chemiluminescent HRP substrate and detected by an imaging system (UVP, BioSpectrum 500). Signal intensities were determined by densitometric analysis using the ImageJ program. The upper right panel shows the calculated rEP amount from the methanol induction for 72 hours. The bottom left panel shows the quantitation of rEP in the Western blotting experiment. M is the protein marker, and P is the synthetic peptide (40 μg/ml) (in the well containing 40 ng of synthetic peptide). 0 indicates not treated with methanol. 3 indicates treatment with methanol for 72 hours. V is the expression vector only without any EP gene insertion as a control.

A1. Cornea irritation score: Degree of opacity

Score	Degree of opacity
0	No ulceration or opacity
1	Scattered or diffuse areas of opacity (other than slight dulling of normal luster), details of iris clearly visible
2	Easily discernible translucent area, details of iris slightly obscured
3	Nacrous area, no details of iris visible, size of pupil barely discernible
4	Opaque cornea, iris not discernible through the opacity

A2. Cornea irritation score: Area of opacity

Score	Area of opacity (AO)
0	0
1	0 < AO < 1/4
2	1/4 < AO < 1/2
3	1/2 < AO < 3/4
4	3/4 < AO

B. Iris irritation score

Score	Iris Damage Value
0	Normal
1	Markedly deepened rugae, congestion, swelling moderate circumcorneal hyperemia, or injection, any of these or combination of any thereof, iris still reacting to light (sluggish reaction is positive)
2	No reaction to light, hemorrhage, gross destruction (any or all of these)

C1. Conjunctiva irritation score: Redness

Score	Redness
0	Normal
1	Some blood vessels definitely hyperemic (injected)
2	Diffuse, crimson color, individual vessels not easily discernible
3	Diffuse beefy red

C2. Conjunctiva irritation score: Chemosis

Score	Chemosis
0	Normal
1	Some swelling above normal (includes nictitating membranes)
2	Obvious swelling with partial evasion of lids
3	Swelling with lids about half-closed
4	Swelling with lids more than half-closed

Supplementary Figure 2. Criteria for the evaluation of eye irritation. Lower conjuctiva sac of the left eye in NZW rabbits were instilled with 0.1 g of piscidin-4-expressing yeast pellet, and irritation scores were assessed at 1, 24, 48 and 72 h after treatment. (A1) Degree of opacity (A2) Area of opacity in Cornea, (B) Iris damage value and (C1) Redness and (C2) Chemosis in Conjuctiva.
